# Myo10-dependent leading-edge coordination maintains physical coherence of migrating dendritic cells

**DOI:** 10.64898/2026.10.17.755813

**Published:** 2026-10-20

**Authors:** Anjelika Gasilina, Paniz Rezvan Sangsari, Natalie M. Badillo, Yang-In Yim, Dillon C. Shrock, Nicole Y. Morgan, John A. Hammer

## Abstract

Dendritic cells (DCs) are the principal leukocytes responsible for priming T cells. DC migration through geometrically complex tissue environments is crucial for initiating robust and spatiotemporally coordinated activation of adaptive immune responses. However, our understanding of the molecular mechanisms that govern physical navigation of DCs remains incomplete. Here we identify the unconventional myosin Myo10 as a critical regulator of DC trafficking. We show that loss of Myo10 attenuates FITC-induced contact hypersensitivity and reduces the efficiency of *in vivo* DC migration and egress from dermal explants but notably has no effect on antigen uptake or *in vitro* T cell priming. We further find that the migration defects of Myo10-deficient cells are exacerbated by increasing geometrical complexity. Mechanistically, Myo10 promotes physical coherence and coordinated leading edge dynamics via its interaction with lamellipodin, which subsequently drives VASP-mediated actin polymerization. Our findings identify a Myo10-lamellipodin-VASP axis critical for leukocyte navigation through restrictive tissue architectures, linking cytoskeletal coordination to effect immune surveillance.

